# Reason and Insanity

**Published:** 1875-12

**Authors:** 


### REASON AND INSANITY.

The dividing line between sanity and insanity, is of so delicate a texture as to leave a very considerable number of persons in the neutral territory. Slight causes are sufficient to take them over the frontier, and hence the necessity of constant vigilance before the point is reached which separates the patient from the common routine of his civil life. In most cases of the kind, it is an error to crowd the subjects together in an atmosphere of insanity, where they have to struggle with a host of adverse influences in their progress towards mental convalescence. The true method of cure is to surrond the patient with persons of sound mind, and this can be done best in the family of the physician. No more effectual medicine can be found than the family life of one of the best educated classes in the community. We should consider fully the agency of physical causes in the production of mental disease. A single timely prescription will often convert the man on the verge of insanity, to his usual serenity of mind. Everybody knows that it is a bad time to ask a favor of a person while he is waiting for his dinner. The impoverished condition of the blood at that moment, causes a nervous irritation, and predisposes to bad temper. Nor should one engage in mental labor soon after the principal meal of the day. The lawyer, with his blood poisoned by the foul stagnant air of city chambers, or the clergyman, wearied with the effort to compose his Sunday sermon, who resumes work immediately after dinner, invites appolexy and sudden death. Not that severe mental labor at the proper season is injurious. A well organized brain demands exercise. The pleasure attendant upon productive brain-work affords an effective protection to the worker. The poet, in the full light of his fancy, refreshes rather than weakens his brain. The orator, who thrills the hearts of the multitude by his impassioned appeals, retires from the triumphant scene, like "a giant refreshed with wine." It is the hard and thankless task-work that frets the fine fabric of the brain, saps the mind of the strong man, and reduces him to the condition of an imbecile. For this reason, probably, among others, diseases of the brain are far more common in this country than in England. The rage for speculation, and the passion for going-ahead, strain the mental fabric to the utmost point. The lesson to be urged, is abstinence from all excesses, the maintenance of a serene and even frame of mind, and moderation in all the physical habits. A healthy brain will be the reward of such a course, and of all temporal prosperity, and even of all spiritual welfare, a healthy brain is the essential condition

---

Borax has many valuable uses in the household. A weak solution snuffed up through the nostrils will relieve catarrh; some cases of sore throat may be cured by using this as a gargle; but it is specially efficacious in allaying inflamation of the membranes lining the cavities of the nose when irritated by cold.

